# Comparison of splenocyte microRNA expression profiles of pigs during acute and chronic toxoplasmosis

**DOI:** 10.1186/s12864-019-5458-y

**Published:** 2019-01-30

**Authors:** Zhaofeng Hou, Dandan Liu, Shijie Su, Lele Wang, Zhenxing Zhao, Yifei Ma, Qiaoqiao Li, Chuanli Jia, Jinjun Xu, Yonghua Zhou, Jianping Tao

**Affiliations:** 1 College of Veterinary Medicine, Yangzhou University, Yangzhou, 225009 People’s Republic of China; 2 Jiangsu Co-innovation Center for Prevention and Control of Important Animal Infectious Diseases and Zoonosis, Yangzhou, 225009 People’s Republic of China; 3 Key Laboratory of Prevention and Control of Biological Hazard Factors (Animal Origin) for Agri-food Safety and Quality, Ministry of Agriculture of China, Yangzhou, 225009 People’s Republic of China; 4 Joint International Research Laboratory of Agriculture and Agri-Product Safety, Yangzhou University, Yangzhou, 225009 People’s Republic of China; 5 Jiangsu Institute of Parasitic Diseases, Wuxi, 214064 People’s Republic of China

**Keywords:** Host regulation, microRNA, Pig, Spleen, *Toxoplasma gondii*, Acute and chronic infection

## Abstract

**Background:**

*Toxoplasma gondii* is an obligate intracellular parasite that infects humans and other warm-blooded animals. Previous quantitative proteomic analyses of infected host cells revealed that the expression of many host proteins is modulated by *T. gondii* infection. However, at present limited data are available on the differentially expressed miRNAs (DEMs) associated with the pathology and host immune responses induced by acute and chronic infection with *T. gondii* in pigs in vivo. In this study, high-throughput sequencing was used to investigate expression profiles of spleen miRNAs at 10, 25 and 50 days post-infection (DPI) in pigs infected with Chinese I genotype strain *T. gondii* isolated from a dead pig.

**Results:**

When compared to the control group, 34, 6 and 86 DEMs were found in spleens of infected pigs at 10, 25 and 50 DPI, respectively. Gene Ontology (GO) enrichment analysis of the target genes of DEMs showed that no GO terms were enriched at 25 DPI, whereas 28 and 241 GO terms, of which two and 215 were sample-specific, were significantly enriched at 10 and 50 DPI, respectively. The top 20 Kyoto Encyclopedia of Genes and Genomes (KEGG) pathways of the target genes of DEMs included signal transduction, immune system, metabolism and diseases. miRNA–gene network analysis revealed that the DEMs played important roles in the host immune response to *T. gondii* infection by modulating expression levels of cellular immunity-related cytokines and immune-related C-type lectins.

**Conclusion:**

Our results not only showed that host miRNA expression is altered by *T. gondii* but also revealed differences in the regulation of key biological processes and pathways involved in host responses to acute versus chronic *T. gondii* infection. This will aid future research into miRNA-target interactions during *T. gondii* infection in pigs and the development of novel therapies against *T. gondii*.

**Electronic supplementary material:**

The online version of this article (10.1186/s12864-019-5458-y) contains supplementary material, which is available to authorized users.

## Background

Toxoplasmosis, caused by *Toxoplasma gondii*, is one of the most prevalent parasitic zoonotic diseases in the world. *T. gondii* infection can cause the acute onset of toxoplasmosis and death in pigs. Ingestion of porcine meat containing persistent tissue cysts is considered to be the major source of *T. gondii* infection in humans [1]. The distribution of *T. gondii* genotypes varies worldwide. The Chinese I genotype strain (ToxoDB #9) is predominant in China [2].

RNA silencing through the action of microRNAs (miRNAs) plays a major role in innate antiviral and antibacterial defenses in plants, insects and animals [3]. Initially reported in *Caenorhabditis elegans* [4], miRNAs are involved in the regulation of gene expression primarily by binding to the 3′ untranslated regions of target mRNAs, where they repress translation or affect the translation process by inducing mRNA cleavage [5]. A growing body of evidence has demonstrated that parasites promote modifications to host miRNAs, underscoring the importance of miRNAs in parasite-host interactions. After invading host cells, parasites may regulate gene expression in target cells [6–9], including specialized immune cells such as macrophages and dendritic cells (DCs) [10], to ensure parasite growth and persistence. Microarray analysis has demonstrated that 24 h post-*T. gondii* infection, over 15% of mRNAs in primary human foreskin fibroblasts display altered abundance relative to uninfected cells [11]. These changes may be explained in part by the differences in host miRNA expression induced by *T. gondii* infection. Zeiner et al. [12] found that miR-17-92 and miR-106b-25, which are known to play crucial roles in apoptosis and G1/S cell cycle transition pathways, were upregulated by the host in response to *T. gondii* infection [13]. Similarly, a subset of miRNA genes, including miR-30c-1, miR-125b-2, miR-23b-27b-24-1 and miR-17-92 cluster genes, are transactivated through promoter binding of STAT3 following *T. gondii* infection in human macrophages [14].

*T. gondii* develops sophisticated strategies to manipulate hosts for efficient intracellular survival [15]. A recent study showed that different *T. gondii* strains differed in their ability to induce host immune responses and signaling pathways [16]. For example, only type II strains were found to upregulate expression of miR-146a, a key immune and inflammatory response regulator. In contrast, another study showed that miR-155 was induced by all *T. gondii* strains assessed [17]. Different *T. gondii* genotypes may also differ in their ability to alter host miRNA expression levels [17].

Studies of the interactions between the host and the Chinese I genotype strain of *T. gondii* are currently very limited. Although alterations to host cells harboring *T. gondii* have been documented at the cellular level, little is known about how the parasite manipulates host cells in vivo. Because the spleen is at the center of humoral and cellular immunity, any functional changes to spleen cells may affect the immune status of the host. Changes in miRNA expression in the spleen following acute *T. gondii* infection have been reported [18]. However, little is known about the status and functions of the spleen during chronic *T. gondii* infection [19]. Information on changes in miRNA expression in the porcine spleen after acute and chronic *T. gondii* infection are also lacking. Therefore, the objective of the present study was to analyze changes in miRNA expression in the porcine spleen after acute and chronic infection with the Chinese I genotype strain of *T. gondii* using next-generation sequencing technology and bioinformatics analysis. Our results provide novel insights into the role of miRNAs in the response to *T. gondii* infection in vivo and will further our understanding of the interactions between the host and *T. gondii.* This will aid future research into the development of novel therapies against *T. gondii*.

## Results

### Clinical symptoms, histopathological examination and PCR detection

**Fig. 1:**
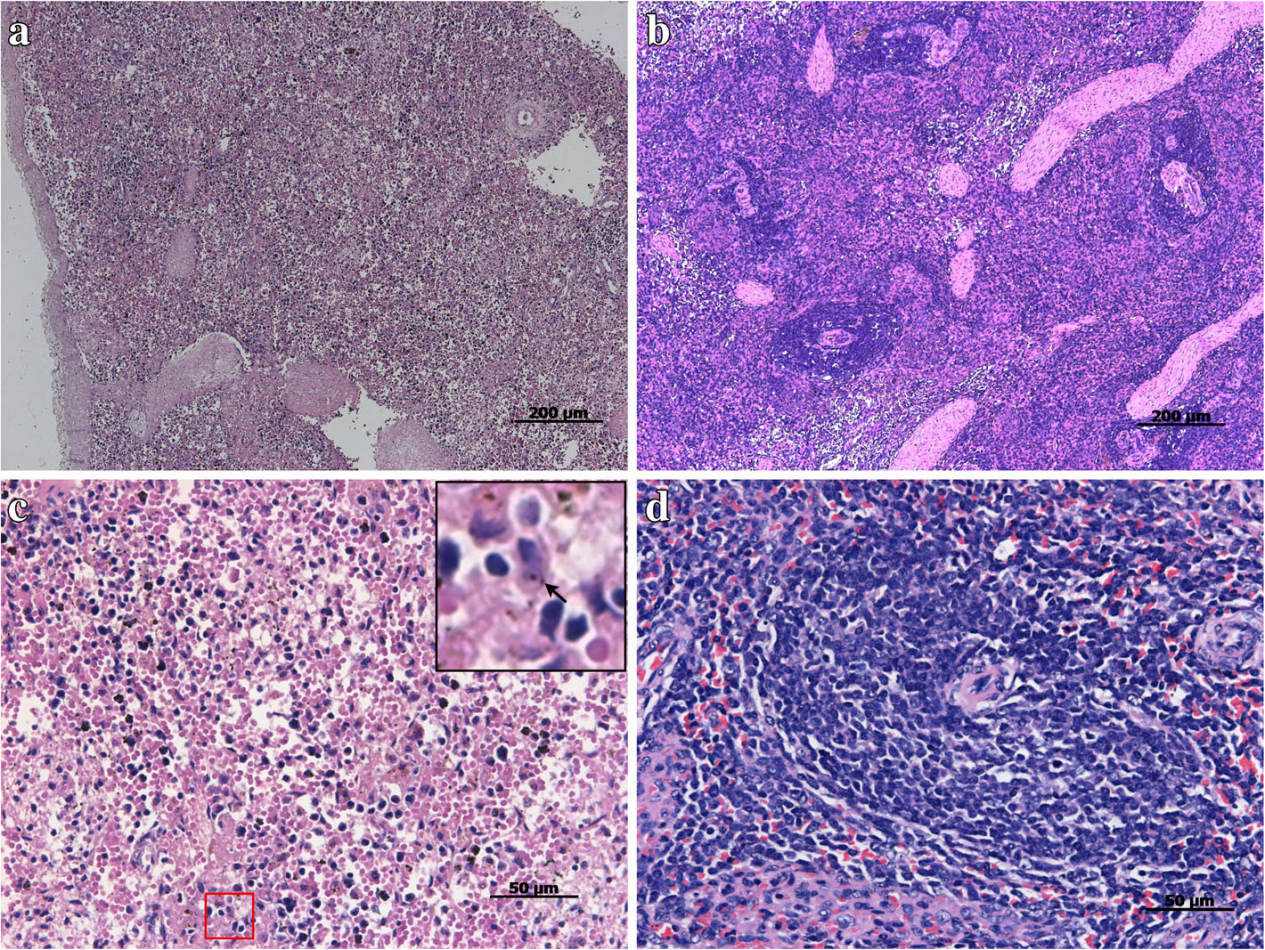
Histopathology of tissue sections prepared from pig spleens by HE staining. **a** and (**c**) represents the spleens prepared from pigs in the infected group. **b** and (**d**) represents the spleens prepared from pigs of the control group. The part marked with a red box in (**c**) was enlarged, and *T. gondii* tachyzoites were indicated with an *arrow*. **a** and (**b**), bar = 200 μm; (**c**) and (**d**), bar = 50 μm

All inoculated pigs developed fever beginning 24 h after inoculation with *T. gondii* and peaking at 3–6 DPI. Fever was accompanied by shivering, anorexia, poor mental state and dyspnea. Three pigs died of *T. gondii* infection between 8 and 10 DPI. The principle lesions observed were severe edema, hemorrhage and necrosis in parenchymatous organs, including brain, lung, liver, lymph nodes and spleen. Microscopic lesions included hepatocellular degeneration, mononuclear cell infiltration and alveolar type II epithelial cell hyperplasia in the lung, damaged and destroyed splenic corpuscles and lymphocytopenia in the spleen (Fig. 1). No clinical symptoms were observed in pigs 20 DPI. No lesions were observed in pigs 25 or 50 DPI.

PCR results showed that all tissues collected at 10 DPI, and brain, lungs and lymph nodes collected at 25 DPI, were *T. gondii*-positive. However at 50 DPI, only brain tissue was positive for *T. gondii* by PCR. *T. gondii* tissue cysts were also found in the brain at this time point by microscopic examination, suggesting that *T. gondii* had established a chronic infection. No clinical symptoms or lesions were observed in the control group at any point during the study.

### Profile of sequencing data

In the infected group, a total of 13,929,425, 13,143,459 and 11,569,642 raw reads were obtained from SI-10, SI-25 and SI-50 sRNA libraries, respectively. In the control group, 14,250,850, 11,741,170 and 13,207,225 raw reads were obtained from SC-10, SC-25 and SC-50 sRNA libraries, respectively. After removing low-quality reads and masking adaptor sequences, clean reads were obtained from the six sRNA libraries (Table 1). The majority of the sRNAs were 20–24 nt in length.

**Table 1:** The list of data filtering (%)

Library	Total reads	N% > 10%	Low quality	5′ contamine	3′ null or insert null	With ployA/T/G/C	Clean reads
SI-10	13,929,425 (100.00)	611 (0.00)	27,246 (0.20)	480 (0.00)	156,647 (1.12)	8824 (0.06)	13,735,617 (98.61)
SI-25	13,143,459 (100.00)	102 (0.00)	11,794 (0.09)	625 (0.00)	317,578 (2.42)	13,064 (0.10)	12,800,296 (97.39)
SI-50	11,569,642 (100.00)	135 (0.00)	10,968 (0.09)	140 (0.00)	120,137 (1.04)	4400 (0.04)	11,433,862 (98.83)
SC-10	14,250,850 (100.00)	530 (0.00)	29,766 (0.21)	695 (0.00)	209,549 (1.47)	28,198 (0.20)	13,982,112 (98.11)
SC-25	11,741,170 (100.00)	104 (0.00)	10,780 (0.09)	481 (0.00)	286,719 (2.44)	10,845 (0.09)	11,432,241 (97.37)
SC-50	13,207,225 (100.00)	559 (0.00)	40,020 (0.30)	993 (0.01)	228,722 (1.73)	5731 (0.04)	12,931,200 (97.91)

**Fig. 2:**
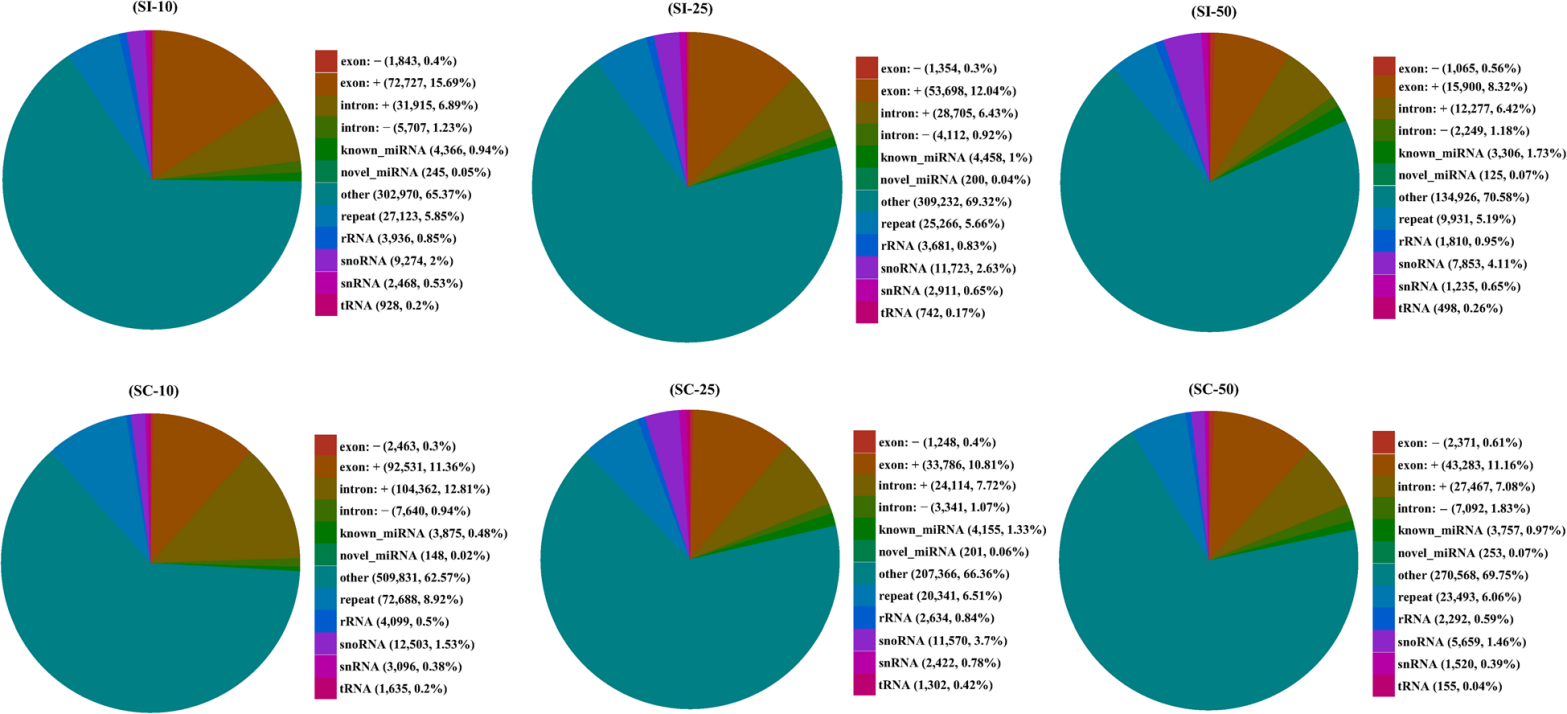
The annotation statistics of the unique reads from samples in each group. ‘exon +’ or ‘exon -’ represents small RNA derived from sense or antisense of exon, respectively; ‘intron +’ or ‘intron -’ represents small RNA derived from sense or antisense of intron, respectively; ‘other’ represents the unannotated small RNA

Between 87.41 and 92.74% of sRNA tags from the six libraries were aligned with the pig genome. Sequences of repeats, exons, introns and other non-coding RNAs, including tRNA, rRNA, snRNA and snoRNA, were successfully annotated. A total of 27.64–36.94% of the reads were unique and unique reads were predominantly exon^+^. Moreover, 0.48–1.73% of the unique reads from each library were identified as known miRNAs and 0.02–0.07% were predicted to be novel miRNAs (Fig. 2). A total of 307 known and 198 novel mature miRNAs, corresponding to 237 and 198 precursors, respectively, were identified with a BLAST search against the miRBase or by recognition of standard stem-loop structures (Table 2). Of these mature miRNAs, 260 known and 25 novel miRNAs were shared amongst the six libraries. Thirteen of the 20 most abundant miRNAs in each sample, including ssc-miR-30a-5p, ssc-miR-143-3p, ssc-let-7a, ssc-miR-26a, ssc-miR-145-5p, ssc-miR-148a-3p, ssc-let-7f, ssc-miR-126-3p, ssc-miR-27b-3p, ssc-miR-21, ssc-let-7i, ssc-let-7 g and ssc-miR-30d were expressed in all six libraries (Additional file 1: Table S1). Of the 307 known and 198 novel mature miRNAs, 309 miRNAs were classified into 157 miRNA families.

**Table 2:** The known and novel miRNAs mapped in pig genome

miRNA	Types	Total	sRNA Libraries					
			SI-10	SI-25	SI-50	SC-10	SC-25	SC-50
Known miRNAs	Mapped mature	307	291	289	279	285	295	282
	Mapped hairpin	291	282	278	273	272	282	277
	Mapped unique sRNA	23,917	4366	4458	3306	3875	4155	3757
	Mapped total sRNA	29,387,126	6,220,575	5,053,714	4,663,061	2,825,377	4,676,267	5,948,132
Novel miRNAs	Mapped mature	198	110	105	68	78	98	121
	Mapped star	53	27	17	17	21	19	27
	Mapped hairpin	212	126	113	79	92	106	129
	Mapped unique sRNA	1172	245	200	125	148	201	253
	Mapped total sRNA	2697	534	422	255	325	450	711

### Differentially expressed MiRNAs in spleens of infected and control pigs at different time points

**Fig. 3:**
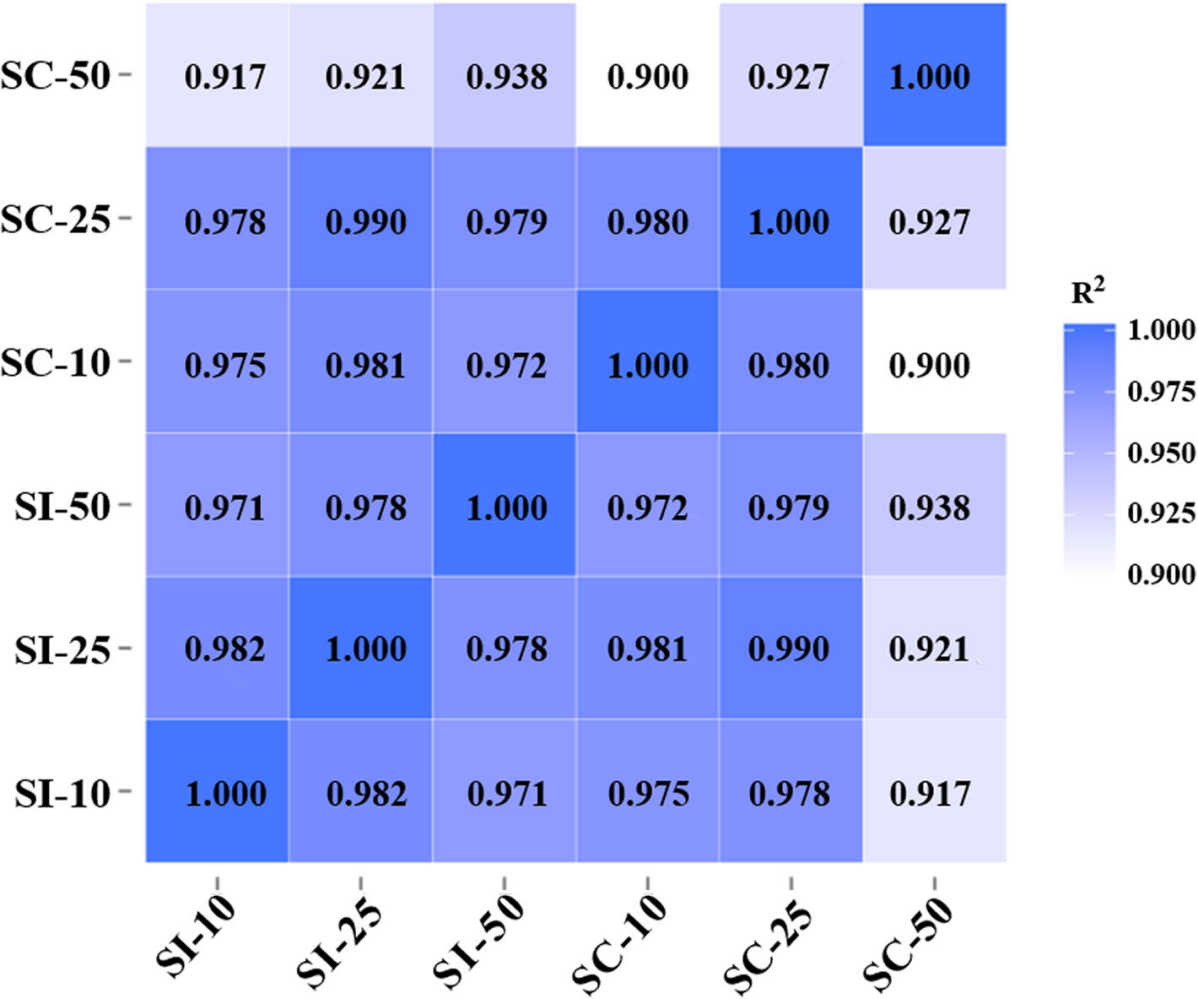
The correlation analysis between samples in different groups. Pearson correlation coefficients were calculated to estimate the association of expression levels between samples

**Fig. 4:**
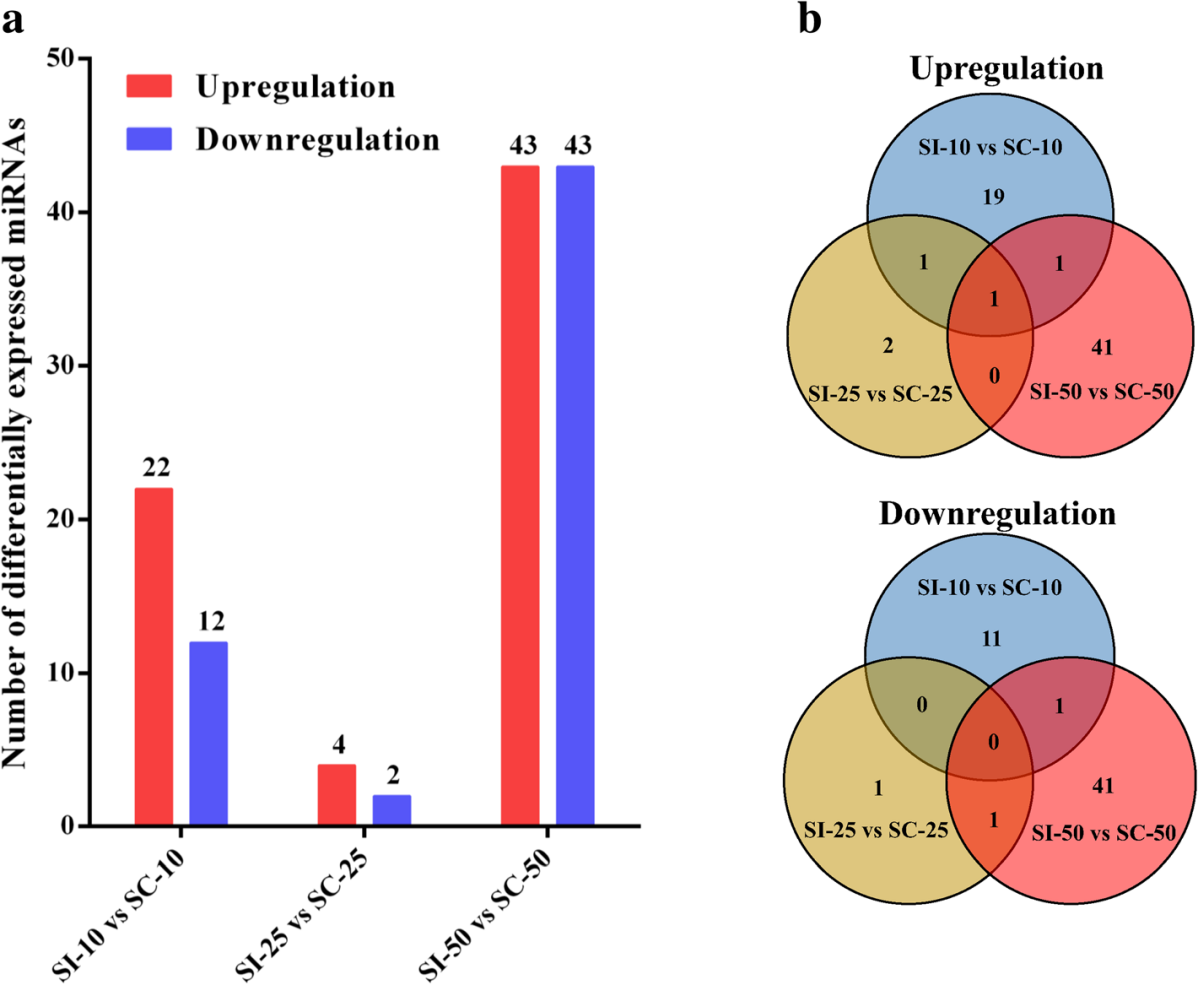
The differential expression analysis of pig spleen miRNAs between the infected and control groups. **a** The number of DEMs between the infected and control groups. **b** The venn diagrams of the DEMs between the infected and control groups

To examine the gene expression patterns of miRNAs in different samples, pearson correlation coefficients were used to estimate expression levels. The correlation coefficients ranged from 0.90 for SC-10 versus SC-50 to 0.99 for SI-25 versus SC-25 (Fig. 3). A total of 109 unique pig-encoded miRNAs were significantly differentially expressed between the infected and control samples at the three time points (*q* < 0.001), including two novel miRNAs from SI-10 (ssc-novel-154 and ssc-novel-882) and one novel miRNA from SI-50 (ssc-novel-262). Compared with the control groups at the same time point, 34, 6 and 86 unique miRNAs were identified as DEMs in SI-10, SI-25 and SI-50 samples, respectively (Fig. 4a; Additional files 2, 3 and 4: Tables S2–S4). A total of 115 DEMs were identified to be sample-specific, including 30 from SI-10, 3 from SI-25 and 82 from SI-50. Of the 69 upregulated DEMs, one (ssc-miR-144) was found in all infected samples. None of the 57 downregulated DEMs were common to all infected samples (Fig. 4b).

### Functional enrichment analysis of target genes of differentially expressed MiRNAs

Candidate target genes of DEMs were predicted using RNAhybrid and miRanda softwares. At 10, 25 and 50 DPI, 838, 25 and 722 target genes were predicted for the upregulated miRNAs and 325, 85 and 2008 target genes were predicted for the downregulated miRNAs, respectively. To better understand the functions of the DEMs, we performed GO and KEGG pathway analysis on these putative target genes.

**Fig. 5:**
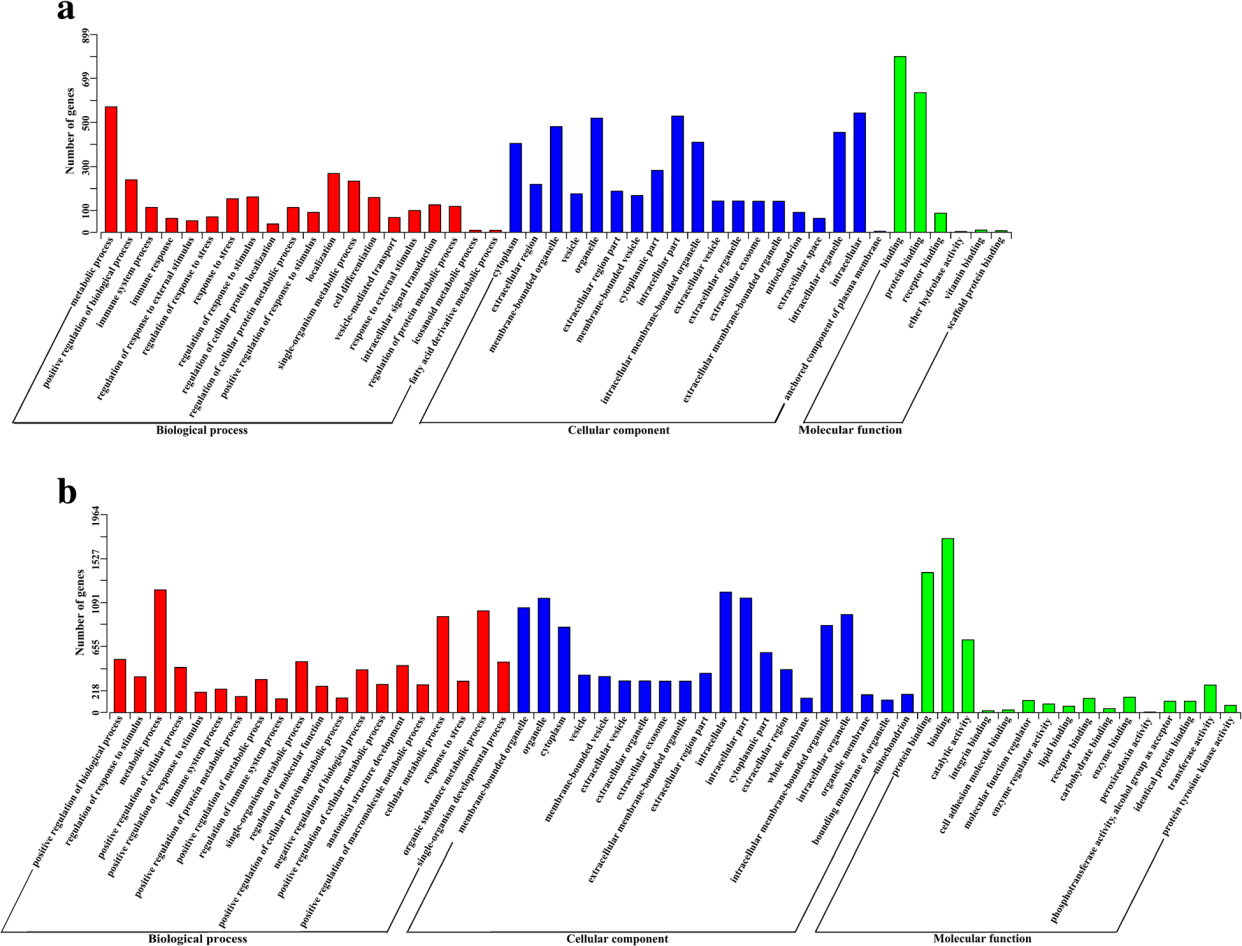
The GO enrichment analysis for target genes of DEMs. **a** The significant enriched GO terms of biological process, cellular component and molecular function for target genes of DEMs at 10 DPI. **b** The significant enriched GO terms of biological process, cellular component and molecular function for target genes of DEMs at 50 DPI

The GO assignments of target genes are shown in Fig. 5. Compared with control samples at the same time points, a total of 28, 0 and 241 significantly enriched GO terms (*P* < 0.01) were identified from SI-10, SI-25 and SI-50 samples, respectively. Of 269 GO terms, 26 were shared between SI-10 and SI-50 samples, most of which were related to cell differentiation, immune responses, localization, metabolic processes, responses to stress and binding. The GO terms receptor binding (GO: 0005102) and vesicle-mediated transport (GO: 0016192), were only found in the SI-10 sample, whereas 215 GO terms, involved to intracellular transport and signal transduction, apoptotic processes, hydrolase activity, metabolic processes and regulation of immune responses, were significantly enriched in the SI-50 sample.

**Fig. 6:**
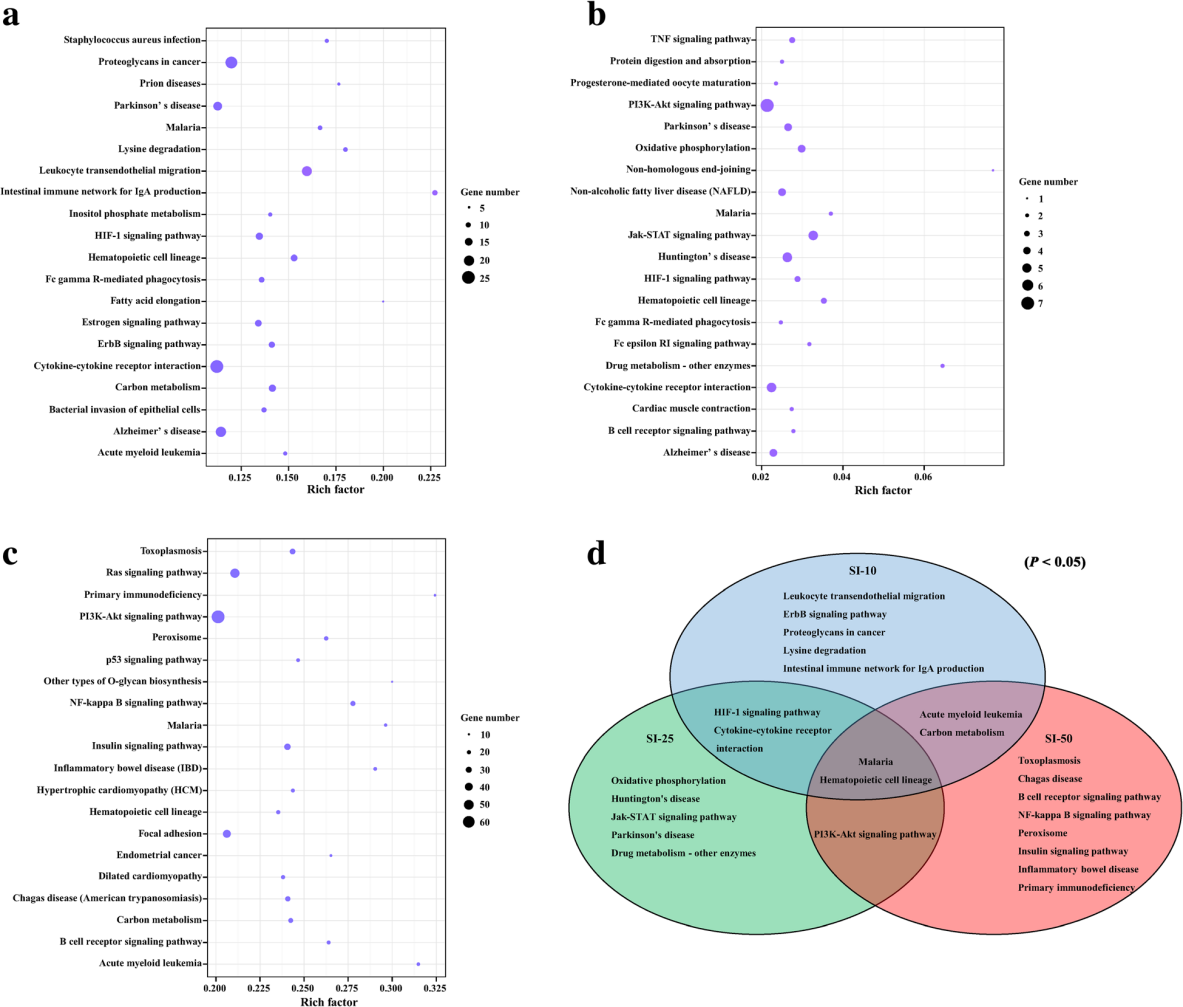
The KEGG enrichment analysis for target genes of DEMs. The top 20 KEGG pathways of differentially expressed miRNAs in pig spleen between the infected and control groups at 10 (**a**), 25 (**b**) and 50 (**c**) DPI. Rich factor indicates the ratio of target genes of DEMs enriched in the pathway among genes annotated in the pathway. **d** The venn diagram of the significant enriched pathways between the infected and control groups (*P* < 0.05)

KEGG pathways with *P* values < 0.05 were analyzed. A total of 255, 121 and 267 KEGG pathways were obtained at 10, 25 and 50 DPI, respectively (Additional file 5: Table S5). The top 20 KEGG pathways of the target genes of DEMs are shown in Fig. 6a, b and c. The most frequently represented pathways were associated with signal transduction, immune system, metabolism (overview, amino acid, energy), and diseases (infectious diseases, cancers, neurodegenerative diseases and immune diseases). KEGG pathway enrichment analysis revealed that 11, 10 and 13 pathways were significantly enriched (*P* < 0.05) at 10, 25 and 50 DPI, respectively. The Venn diagram of enriched pathways at the three time points is shown in Fig. 6d. The pathways “Leukocyte transendothelial migration” (ssc04670) and “Intestinal immune network for IgA production” (ssc04672) were found only at SI-10, whereas more immune-related pathways, including “B cell receptor (BCR) signaling” (ssc04662), “Inflammatory bowel disease” (ssc05321), “NF-kappa B signaling” (ssc04064) and “Primary immunodeficiency” (ssc05340) pathways were found only at SI-50. The pathways “Malaria” (ssc05144) and “Hematopoietic cell lineage” (ssc04640) were common to all three time points, indicating that both pathways play important regulatory roles during *T. gondii* infection.

### MiRNA–gene network analysis

**Fig. 7:**
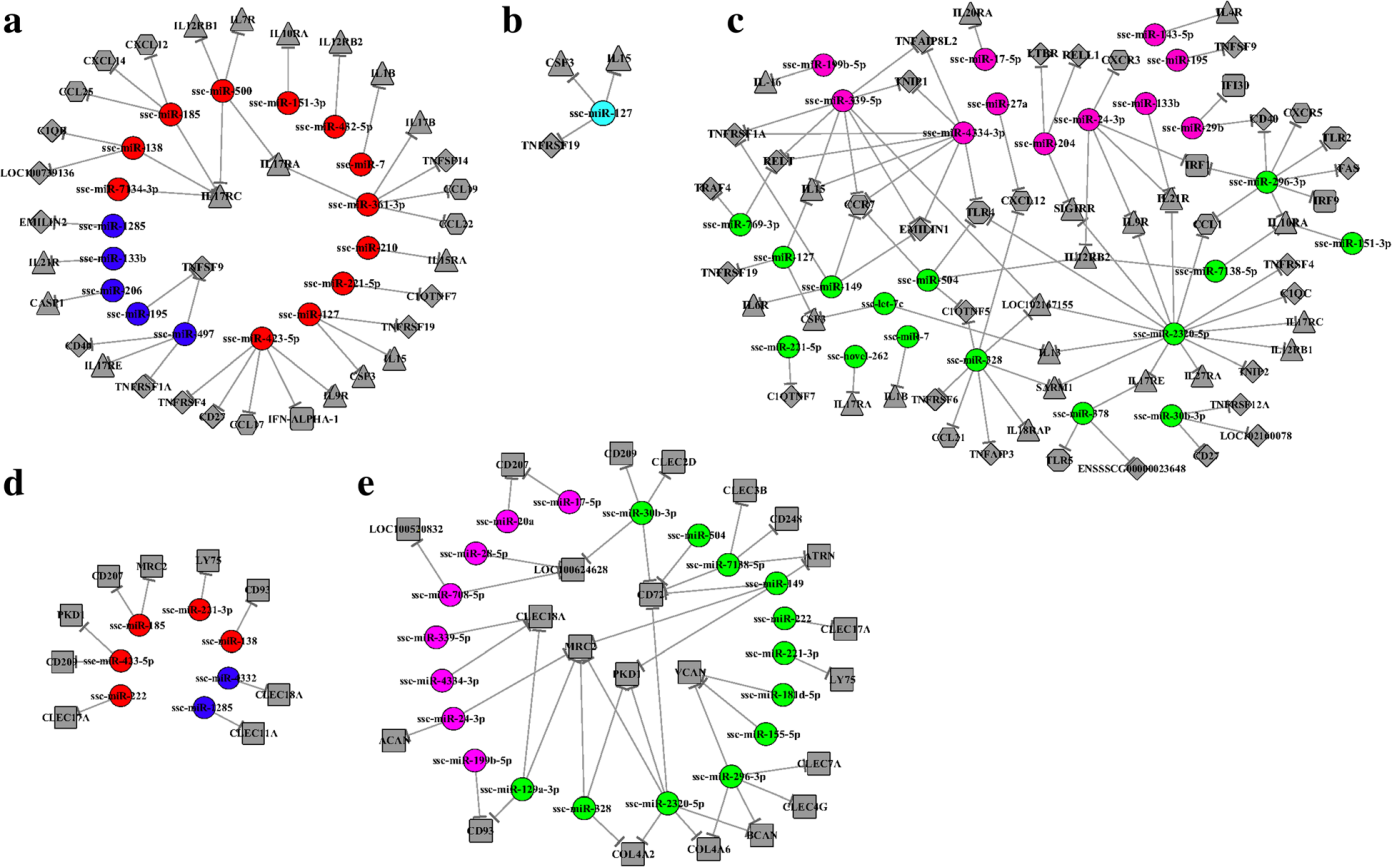
The network analysis of the interaction between the DEMs and their potential target genes. The node shapes were used for representing the different miRNAs or target genes, which were connected by edges (negative interaction between miRNA to target gene). And the colours of spherical nodes were represented the upregulated miRNAs or downregulated ones in porcine spleen at different time points. **a**, **b** and **c** The interaction between the cytokines and the DEMs in pig spleens at different time points after *T. gondii* infection. **d** and **e** The interaction between the C-type lectins and the DEMs in pig spleens

Regulatory networks were constructed for the immune-related target genes and DEMs at 10, 25 and 50 DPI (Fig. 7). At 10 DPI, 33 different cytokines, including chemokines, interleukins (ILs), tumor necrosis factors (TNF) and interferons (IFN), were potentially regulated by 17 DEMs. Among these DEMs, ssc-miR-361-3p and ssc-miR-423-5p regulated the greatest number of target genes, with five targets each. The target gene regulated by the greatest number of DEMs was *IL17RC*, with four miRNAs, followed by *TNFSF9* and *IL17RA*, each regulated by two DEMs (Fig. 7a). These results indicate that these DEMs and cytokines might play important roles in the host response to acute *T. gondii* infection. At 25 DPI, one miRNA, ssc-miR-127, was found to regulate the expression of three cytokine genes, *TNFRSF19*, *IL15* and colony stimulating factor 3 (*CSF3*) (Fig. 7b). At 50 DPI, 56 different cytokines were found to be regulated by 26 DEMs. Of these, ssc-miR-2320-5p had the greatest number of target genes, with 15 targets, followed by the miRNAs ssc-miR-328, ssc-miR-296-3p, ssc-miR-339-5p and ssc-miR-4334-3p, which each had eight targets. The target gene regulated by the greatest number of DEMs was *CCR7*, with four miRNAs. Nine other target genes were each regulated by three DEMs (Fig. 7c). These cytokines may play key roles in the interaction between host cells and *T. gondii* during chronic infections.

Many genes encoding C-type lectins were also found to be targets of host miRNAs induced by *T. gondii*. The interaction networks between these C-type lectins and the identified miRNAs were established at the different time points. At 10 DPI, nine candidates containing C-type lectin domains were found to be regulated by seven DEMs. The miRNAs ssc-miR-185 and ssc-miR-423-5p were shown to regulate the greatest number of target genes, with two targets each (Fig. 7d). At 50 DPI, a total of 22 C-type lectins were regulated by 20 DEMs. The ssc-miR-2320-5p was determined to have the greatest number of target genes, with six targets), followed by ssc-miR-296-3p, which had five targets. Of the 22 C-type lectin genes, *CD72* and *MRC2* were regulated by the greatest number of DEMs, with five miRNAs each (Fig. 7e).

### RT-qPCR validation

**Fig. 8:**
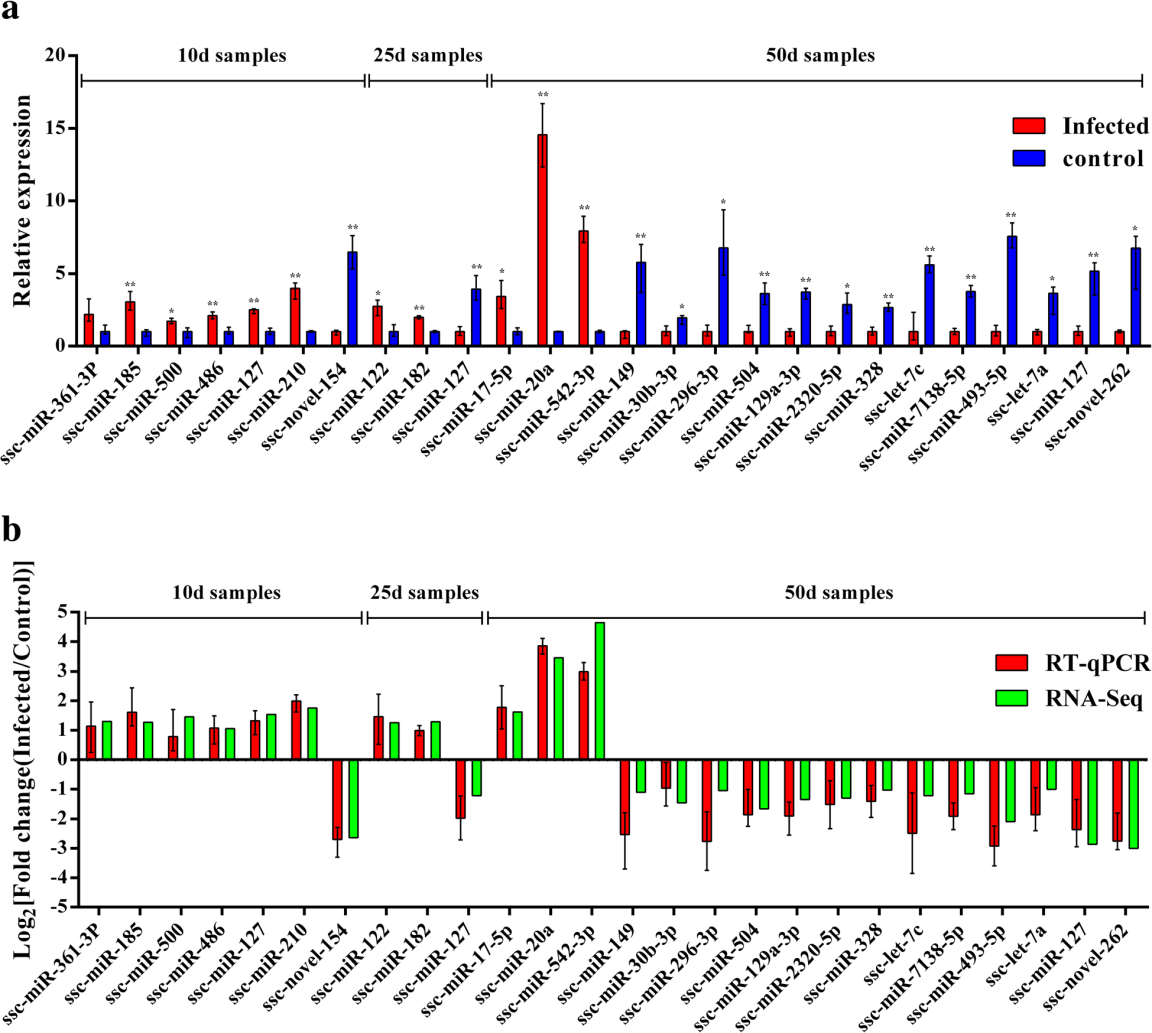
Sequencing data validated by RT-qPCR. **a** The expression level of differentially expressed miRNAs validated by RT-qPCR. MicroRNA expression was quantified relative to the expression level of U6 using the comparative cycle threshold (ΔCT) method. * *P* < 0.05, **P < 0.01. **b** Comparison of the expression pattern of the sequencing data and RT-qPCR data. Log_2_ (fold change) > 0 indicates the transcript upregulated in infection group compared to the control group. Log_2_ (fold change) < 0 indicates the transcript downregulated in infection group compared to the control group

Twenty-six representative DEMs, including a miRNA (ssc-miR-127) with different expression patterns at the three time points, were validated with quantitative reverse transcription real-time PCR (RT-qPCR). miRNA sequences in which uracil was replaced by thymine were used as the forward primers for the real-time PCR described in Table 3. With the exception of ssc-miR-182, ssc-miR-542-5p and ssc-novel-154, the miRNAs assessed with RT-qPCR were identified as having roles in the regulation of protein expression in the specific signaling pathways or miRNA–gene network analysis performed in this study. For example, ssc-miR-122 and ssc-miR-127 were associated with the host inflammatory response; ssc-miR-361-3p, ssc-miR-500, ssc-miR-486 and ssc-miR-185 were involved in immune cell differentiation; ssc-miR-328, ssc-let-7c, ssc-miR-493-5p, ssc-let-7a and ssc-miR-7138-5p were related to B cell ontogeny and production of protective antibodies; ssc-miR-30b-3p, ssc-miR-504, ssc-miR-149, ssc-miR-296-3p, ssc-miR-129a-3p and ssc-miR-2320-5p were found to be involved in host protective responses and parasite growth. With the exception of that of ssc-miR-361-3p, the expression patterns of miRNAs measured with RT-qPCR corresponded with those obtained by high-throughput sequencing (Fig. 8), confirming the accuracy and reliability of sequencing results used for functional analyses.

**Table 3:** The sequences of miRNAs used for RT-qPCR validation

miRNAs	Sequences
ssc-miR-361-3p	CCCCCAGGUGUGAUUCUGAUUUGC
ssc-miR-185	UGGAGAGAAAGGCAGUUCCUGA
ssc-miR-500	AUGCACCUGGGCAAGGAUUCU
ssc-miR-486	UCCUGUACUGAGCUGCCCCGAG
ssc-miR-127	UCGGAUCCGUCUGAGCUUGGCU
ssc-miR-210	CUGUGCGUGUGACAGCGGCUGA
ssc-novel-154	GUGGUUAGUACUCUGCCUUA
ssc-miR-122	UGGAGUGUGACAAUGGUGUUUGU
ssc-miR-182	UUUGGCAAUGGUAGAACUCACACU
ssc-miR-17-5p	CAAAGUGCUUACAGUGCAGGUAG
ssc-miR-20a	UAAAGUGCUUAUAGUGCAGGUA
ssc-miR-542-3p	UGUGACAGAUUGAUAACUGAAA
ssc-miR-149	UCUGGCUCCGUGUCUUCACUCCC
ssc-miR-30b-3p	CUGGGAGGUGGAUGUUUACUU
ssc-miR-296-3p	AGGGUUGGGCGGAGGCUUUCC
ssc-miR-504	AGACCCUGGUCUGCACUCUAUCU
ssc-miR-129a-3p	AAGCCCUUACCCCAAAAAGCAU
ssc-miR-2320-5p	UGGCACAGGGUCCAGCUGUCGG
ssc-miR-328	CUGGCCCUCUCUGCCCUUCCGU
ssc-let-7c	UGAGGUAGUAGGUUGUAUGGUU
ssc-miR-7138-5p	UCCCAGCAAGUGUCCAUCCAUCU
ssc-miR-493-5p	UUGUACAUGGUAGGCUUUCAUU
ssc-let-7a	UGAGGUAGUAGGUUGUAUAGUU
ssc-novel-262	UGAGCCACAGAAACUCCAGGACU

## Discussion

*T. gondii* infection is mainly acquired by ingestion of food or water that is contaminated with oocysts shed by cats or by eating undercooked or raw meat containing tissue cysts. Under natural infection conditions, sporozoits and bradyzoites enter all nucleated cells including enterocytes by active penetration and form a cytoplasmic vacuole. After repeated replication, host cells are disrupted and tachyzoites are disseminated via the bloodstream and infect many tissues including spleen [20]. So the intestinal mucosa is the first protective barrier of the host. It has been observed that the enterocytes invaded by *T. gondii* can induce the activation of lymphocytes, monocytes, macrophages and dendritic cells in the lamina propria of intestinal mucosa to resist and eliminate the parasites [21, 22]. In our original experimental design, we planned to infect pigs with *T. gondii* oocysts. But unfortunately, no *T. gondii* oocysts were found from cats infected with *T. gondii* YZ-1 strain. And due to mouse-virulent of *T. gondii* YZ-1 strain, no tissue cysts were obtained from mice infected with tachyzoites [23]. Therefore, the pigs in this study were infected intravenously with *T. gondii* tachyzoites. Compared to natural infection, *T. gondii* tachyzoites could directly reach the spleen with blood flow, invade spleen cells, and rapidly induce immune responses of the host. And thus, some miRNAs from pig infected intravenously with *T. gondii* tachyzoites would probably be omitted.

In this study, 109 DEMs were identified in porcine spleen samples at three time points after *T. gondii* infection. Of these, 86 DEMs were found at 50 DPI. This result is consistent with previous studies in mice that found that more miRNAs were differentially expressed at 21 DPI than at 14 DPI [24] and that chronic infection induced a greater number of differentially expressed genes [25]. Some DEMs, including miR-21, miR-142-3p, miR-101, miR-184, miR-182, miR-155, miR-181a, miR-146, miR-126, miR-29, miR-17 and miR-19, are known to be involved in the host immune response [26, 27]. For example, it has been reported that the downregulation of miR-184 and miR-16, which were downregulated at 10 DPI in the present study, prevents hyperactivation of T cells and macrophages [28]. Deficiency in miR-146, similar to miR-146b, which was upregulated at 10 and 25 DPI in this study, in regulatory T cells results in dysregulation of IFN-γ responses. The upregulation of miR-182, which we observed at 25 DPI, has been associated with the expansion of T helper cells [29]. miR-181d and miR-155, which were downregulated at 50 DPI in this study, are known to be involved in T cell development and differentiation, whereas miR-17 upregulation has been associated with B cell generation [30]. miR-21, which we found was upregulated at 50 DPI, acts as a negative regulator of toll-like receptor (TLR) 4 and inhibits the expression of pro-inflammatory cytokines [31]. miR-126, which was upregulated at 50 DPI in our study, is a potent and specific activator of T helper 2-regulated allergic inflammatory responses [32]. Depletion of miR-146a, downregulated at 50 DPI in the present study, has been implicated in hyperactive responses to infection [33]. These reports indicate that the DEMs induced by acute *T. gondii* infection in our study are mainly related to the activation of immune cells, whereas those induced by chronic infection are mainly involved in the inhibition of inflammatory response. Furthermore, some of the DEMs identified in this study have also been found in mice and cats infected with *T. gondii*. In the murine model, miR-155-5p and miR-146a were upregulated within 48 h of infection with type II strain [17]. However, in the present study the expression levels of both of these miRNAs did not differ during acute infection and were downregulated during chronic infection. A large number of the DEM homologs identified in this study have also been found in the livers of domestic cats following acute *T. gondii* infection [34] and the expression patterns of several homologs, including miR-361-3p, miR-151-3p and miR-30b-5p, in the acute phase were consistent with previous reports, indicating that these miRNAs are induced in by *T. gondii* infection in different hosts and have important roles during acute infection.

In KEGG enrichment analysis of the target genes in this study, two enriched pathways, malaria and hematopoietic cell lineage, were identified at 10, 25 and 50 DPI (Fig. 6d). The malaria pathway includes processes involved in the invasion of hepatocytes by malaria sporozoites, subsequent infection of red blood cells by merozoites and the eventual activation of the host immune response. Therefore, our results support the findings of a previous study that found that the process of host cell invasion is very similar between *T. gondii* and *Plasmodium* [35]. The hematopoietic cell lineage pathway includes processes involved in stem cells differentiation into several different cell types [36, 37]. In the present study, at 10 DPI the target genes belonging to the hematopoietic cell lineage pathway were mainly related to the regulation of initial T cell differentiation and to myeloid-related DC and macrophage generation (Additional files 6 and 7: Figure S1, Table S6). Inhibition of the process of host T cell differentiation by the miRNAs ssc-miR-361-3p, ssc-miR-500, ssc-miR-486 and ssc-miR-185 (Additional file 2: Table S2; Fig. 8) may downregulate host immune responses, which may inhibit damage caused by excessive immunity and create an immune environment that is conducive to the survival of parasites. At 25 DPI, erythropoietin (*EPO*) and *CSF3*, target genes belonging to the hematopoietic cell lineage pathway, were regulated by ssc-miR-122 and ssc-miR-127, respectively (Additional file 3: Table S3; Fig. 8). EPO has been found to regulate erythrocyte production and cytokine formation [38] and CSF3 has been associated with neutrophil production. Thus, the host inflammatory response could be regulated by altering *EPO* and *CSF3* expression. At 50 DPI, we identified 23 DEMs associated with target genes involved in the differentiation of several different immune cell types. For example, the downregulation of ssc-miR-328, ssc-miR-2320-5p and ssc-miR-30b-3p (Additional file 4: Table S4; Fig. 8) may induce B cell proliferation in the spleen, resulting in the production of protective antibodies against chronic *T. gondii* infection (Additional files 6 and 7: Figure S1, Table S6).

Infection stage-specific pathways were found at three time points after *T. gondii* infection. At 10 DPI, leukocyte transendothelial migration (ssc04670), intestinal immune network for IgA production (ssc04672), proteoglycans in cancer (ssc05205), ErbB signaling (ssc04012) and acute myeloid leukemia (ssc05221) pathways were enriched. ssc04670 and ssc04670 are immune-related pathways, whereas ssc05205, ssc04012 and ssc05221 are cancer-related pathways that were also found to be enriched in the spleens of mice affected by acute *T. gondii* infection [18]. The enrichment of these pathways suggests that the host immune system and cancer processes are activated by *T. gondii* infection. At 25 DPI, two neurodegenerative disease pathways, Huntington’s disease (ssc05016) and Parkinson’s disease (ssc05012), were enriched (Fig. 7), suggesting that the host’s central nervous system is damaged when parasites invade host brain cells. A recent study in mice showed that *T. gondii* loads in the brain increased until 21 DPI and persisted at high levels throughout the infection [39].

More stage-specific pathways were found to be enriched at 50 DPI than at 10 and 25 DPI. Chagas disease (ssc05142) and toxoplasmosis (ssc05145) were two enriched pathways related to protozoan disease. The DEMs in the toxoplasmosis pathway were mainly involved in the regulation of the expression of genes related to anti-inflammatory responses and cell survival, whereas the DEMs in the Chagas disease pathway were mainly involved in the apoptosis of infected cells and the intracellular persistence of parasites. The downregulation of miRNAs in the Chagas disease pathway, including ssc-miR-30b-3p, ssc-miR-504, ssc-miR-149, ssc-miR-296-3p, ssc-miR-129a-3p and ssc-miR-2320-5p (Additional file 4: Table S4; Fig. 8), may contribute to the inhibition of host protective responses and the promotion of parasite growth (Additional files 8 and 9: Figure S2, Table S7), indicating that these miRNAs may be associated with parasite immune evasion mechanisms. The BCR signaling pathway (ssc04662), which is mainly involved in B cell ontogeny and immunoglobulin production, was another important stage-specific pathway enriched at 50 DPI. Nine miRNAs involved in this pathway, including ssc-miR-149, ssc-miR-328, ssc-let-7c, ssc-miR-493-5p, ssc-let-7a, ssc-miR-30b-3p, ssc-miR-2320-5p, ssc-miR-504 and ssc-miR-7138-5p, were identified in this study (Additional file 4: Table S4; Fig. 8). ssc-miR-149 downregulation may directly promote immune responses by regulating C-JUN expression (Additional files 10 and 11: Figure S3, Table S8). Abnormal B cell signaling has been linked to various diseases, including lupus, lymphoma and other immune disorders [40], suggesting that abnormalities in the BCR signaling pathway induced by miRNAs may contribute to the pathogenesis associated with chronic toxoplasmosis infection.

Analysis of miRNA–gene networks between immune-related target genes and DEMs revealed that target genes encoding TNFRSF4, C1QTNF7, CD27, IL1B, IL10RA, IL12RB1, IL17RA, IL17RC, CSF3 and TNFSF9 were all upregulated in SI-10 samples and downregulated in SI-50 samples, with the exception of TNFSF9 (downregulated in SI-10 samples and upregulated in SI-50 samples) (Fig. 7a and c). TNFRSF4 (OX40, CD134) and its binding partner are members of the TNFR/TNF superfamily and are involved in the control of inflammatory diseases and cancer [41]. CD27, a member of the TNF-receptor superfamily, is required for the maturation of conventional natural killer (NK) cells and the generation and long-term maintenance of T cell immunity and plays a key role in regulating B cell activation and immunoglobulin synthesis [42, 43]. IL-1β, IL-12, IL-17 and their receptors have also been shown to be important mediators of the inflammatory response [44–46] and play key roles in host immune responses induced by *T. gondii* infection [47]. Inhibition of IL-12 production is an important part of the immune evasion mechanism of *T. gondii* [48]. TNFSF9 was confirmed to play important roles during NK and B cell activation [49, 50]. Therefore, the differences in the expression patterns of cytokines regulated by miRNAs that we observed at 10 and 50 DPI may be due to differences in the host immune responses induced by acute and chronic *T. gondii* infections. Interestingly, only three cytokines were found to be involved in host immune regulation at 25 DPI (Fig. 7b). This seems to suggest that this time point may be a special phase of *T. gondii* infection.

C-type lectins, membrane-related receptors involved in the presentation of antigens, have been found to play important roles in *Trypanosoma cruzi*, *Leishmania* and *Neospora caninum* infections [51, 52]. In the present study, we found that a number of C-type lectins were regulated by miRNAs in SI-10 and SI-50 samples. Analysis of miRNA-gene networks between C-type lectins and DEMs showed that genes encoding CD209 (DC-SIGN), PKD1, CLEC17A and lymphocyte antigen 75 (LY75) were upregulated in SI-10 samples but were downregulated in SI-50 samples (Fig. 7d and e). Previous research has reported that the expression of CD209 on macrophages activates phagocytosis, mediates DC rolling interactions with endothelium, activates CD4^+^ T cells and initiates innate immunity by modulating TLRs [53, 54]. LY75 expressed on CD8^+^ DCs plays a role in antigen processing and presentation in the context of both MHC class I and class II molecules and generates T helper 1-mediated immune responses through an IL-12-independent and CD70-dependent mechanism [55]. The roles of those C-type lectins need to be confirmed in *T. gondii* infection. However, C-type lectins regulated by miRNAs clearly play important roles in both acute and chronic *T. gondii* infection in vivo.

## Conclusions

To our knowledge, this is the first report on miRNA expression profiles in the spleens of pig infected with *T. gondii*. A total of 109 DEMs, many of which are known to be involved in the host immune response, were found in pig spleens following *T. gondii* infection. Compared to 10 DPI (34 DEMs) and 25 DPI (6 DEMs), more differences in the expression of host miRNAs were found between the infected and control groups at 50 DPI (86 DEMs). GO and KEGG enrichment analyses suggested that *T. gondii* infection may modulate different host biological processes through DEMs. miRNA–gene network analysis revealed differences in the host immune response to acute and chronic *T. gondii* infection. This study provides a foundation for research on miRNA-target interactions during *T. gondii* infection in pigs. Furthermore, the findings of this study will contribute to the elucidation of the mechanisms responsible for susceptibility and to spleen pathophysiology of infection in other organisms, including humans.

## Methods

### Animals and experimental infection

A YZ-1 strain of *T. gondii*, obtained from a home-bred wild boar in Jiangsu Province, China in 2011, was cryopreserved in liquid nitrogen in our laboratory. The strain was confirmed to be ToxoDB PCR-RFLP genotype #9 (Chinese I) and shown to be virulent in mice in our previous study [23]. Twenty-four 5-week-old commercial pigs were provided by the Jiangsu Meilin Animal Husbandry Co., Ltd. (Jiangsu, China), and proven to be free from *T. gondii*, classical swine fever virus, porcine reproductive and respiratory syndrome virus, porcine pseudorabies virus, porcine parvovirus and porcine circovirus virus by specific IgM and IgG antibody tests using ELISA and the whole blood nucleic acid detection using PCR methods. All pigs were housed individually in standard breeding houses for 1 week prior to infection. All pigs were fed the same commercial food.

Pigs were randomly divided into the control group (9 pigs) or the infection group (15 pigs). Each pig in the infection group was intravenously injected with 5 × 10^7^ *T. gondii* tachyzoites. Pigs in the control group were injected with phosphate buffered saline (PBS) instead of tachyzoites. In addition, five ICR mice purchased from the Comparative Medicine Center of Yangzhou University (Yangzhou, China) were inoculated intraperitoneally with 200 tachyzoites to assess *T. gondii* viability.

### Clinical symptoms, histopathological examination and PCR detection

After infection, clinical symptoms were observed and rectal temperatures were measured twice daily. At 10, 25 and 50 days post-infection (DPI), three pigs from each group were sacrificed under deep anesthesia with sodium pentobarbital (200 mg/kg body weight) by captive bolt for small animals. The brain, liver, spleen, lungs and lymph nodes of pigs were collected for histopathological observation and for *T. gondii* detection using two PCR methods targeting the 529-bp repetitive sequence (AF146527) and B1 gene (AF179871) as previously described [56, 57], respectively. Each reaction comprised 1 × PCR Buffer (100 mM Tris-HCl, pH 8.3, 500 mM KCl, 15 mM MgCl_2_), 0.2 mM dNTP, 0.4 μM of each primer, 0.5 U of *Taq* DNA polymerase, and 2 μL of template to a total volume of 50 μL. The conditions for amplification were as follows: 95 °C for 4 min, 35 cycles of 94 °C for 30 s, 58 °C (60 °C for B1 gene) for 40 s, and 72 °C for 1 min, and a final step of 72 °C for 10 min. The genomic DNA of the YZ-1 strain was set as the positive control, whereas the tissue DNA of a pig, confirmed *T. gondii* infection-free by PCR, was the negative control for each reaction.

### Sample collection and RNA extraction

Spleens collected from pigs were thoroughly rinsed in PBS and immediately frozen in liquid nitrogen. The spleen samples were then stored at − 80 °C until RNA extraction.

Total RNA was prepared from individual spleen samples using TRIzol Reagent (Invitrogen, Carlsbad, CA, USA) according to the manufacturer’s protocol. The quality and quantity of RNA samples were assessed using an Agilent 2100 Bioanalyzer (Agilent Technologies, Santa Clara, CA, USA) and a Nanodrop 2000 (Thermo Scientific, Wilmington, MA, USA), respectively.

### Small RNA library preparation and sequencing

Three individual RNA samples with high RNA integrity values from each group at each of the time points were equally pooled. A total of 3 μg total pooled RNA was used as input material for the small RNA (sRNA) libraries. Thus, a total of six sRNA libraries were generated, including SI-10, SI-25 and SI-50 from samples collected from the infection group at 10, 25 and 50 DPI, respectively, and SC-10, SC-25 and SC-50 from samples collected from the control group at 10, 25 and 50 DPI, respectively. Libraries were generated using NEBNext® Multiplex Small RNA Library Prep Set for Illumina® (NEB, Ipswich, MA, USA) following the manufacturer’s recommendations and sequenced at the Novogene Bioinformatics Institute (Beijing, China) on an Illumina Hiseq 2500 platform (Illumina, San Diego, CA, USA) following the vendor’s instructions.

After sequencing, raw reads were processed using custom PERL and Python scripts. The sequencing error rate of each base was evaluated using the Phred score [58]. Clean reads were obtained by removing reads containing poly-N, reads with 5′ adapter contaminants, reads without the 3′ adapter or insert tags, reads containing poly A, T, G or C and low-quality reads from raw data. In addition, reads shorter than 18 nt or longer than 35 nt were discarded.

### Read mapping and annotation

All filtered sRNA tags were aligned onto the pig genome with Sscrofa 10.2 from Ensembl (http://www.ensembl.org/Sus_scrofa/Info/Index) using Bowtie without mismatch to analyze their expression and distribution [59].

To remove tags originating from protein-coding genes, repeat sequences, rRNA, tRNA, snRNA and small nucleolar RNA (snoRNA), sRNA tags were mapped to RepeatMasker (http://www.repeatmasker.org/), the Rfam database (http://rfam.xfam.org/). Known porcine miRNAs were then identified from the mapped sRNA tags using miRBase 20.0 (http://www.mirbase.org/). miREvo [60] and miRDeep 2 [61] were used to predict novel miRNAs by examining secondary structures. Custom scripts were also used to obtain the miRNA counts and the base bias on the first position of identified miRNAs of certain lengths and on each position of all identified miRNAs. In addition, miRNAs that might have base edits were detected by aligning all the sRNA tags to mature miRNA sequences, allowing a maximum of one mismatch. Furthermore, an exact match to highly conserved seed regions (positions 2–7 of the mature miRNA) [5] was used to classify different miRNA families. And families of mapped miRNAs were identified from other species using Rfam (http://rfam.xfam.org/search).

Full details of the sequence data were submitted to the Gene Expression Omnibus (GEO) public database (http://www.ncbi.nlm.nih.gov/geo/) with the GEO accession number GSE113130. The raw data are available in the NCBI Sequence Read Archive under the accession number PRJNA450089.

### Analysis of differentially expressed MiRNAs

miRNA expression levels were estimated by the number of transcripts per million clean tags (TPM) [62]. Differential expression analysis between groups was performed using the R package DEGseq (2010). *P* values were adjusted using *q* values [63]. The *q* value < 0.01 and |log_2_(fold change)| > 1 were set as the threshold values for differential expression.

### Predicted target genes of MiRNAs and bioinformatics analysis

To understand the cellular functions of DEMs and to identify their associated molecular pathways, target genes of DEMs were predicted using miRanda [64] and RNAhybrid [65]. GO (http://www.geneontology.org/) enrichment analysis of DEMs was performed using the R package GOseq with a gene length bias correction [66]. GO terms with a *P* value < 0.01 were considered significantly enriched with DEMs. Additionally, pathway analysis was used to classify DEMs into significantly enriched pathways (*P* < 0.05) according to KEGG (http://www.genome.jp/kegg/). We used KOBAS software for statistical tests of the enrichment of DEMs in KEGG pathways [67]. In addition, some DEMs-regulated pathways were analyzed by the KEGG map annotations [68, 69].

### MiRNA–gene networks

To gain insight into the interactions between miRNAs and target genes related to the immune response against *T. gondii* and to study the relationships between DEMs at different time points, miRNA-gene networks were created and visualized using Cytoscape 3.4.0 software [70].

### Quantitative real-time PCR validation

To verify sequencing results, 26 DEMs, including 24 known and two novel miRNAs, were selected and measured using SYBR green-based RT-qPCR. miRNA sequences and primers were synthesized by BGI Co. Ltd. (Shenzhen, China). Total RNA extracted from spleen samples was reverse transcribed into cDNA using a Mir-x™ miRNA First-Strand Synthesis and SYBR® RT-qPCR Kit (TaKaRa, Dalian, China) following the manufacturer’s instruction. Primers were used at 10 μM each to amplify target miRNAs in a 20 μl reaction mixture. RT-qPCR cycling conditions were as follows: 95 °C for 5 min; followed by 40 cycles of 95 °C for 5 s, 60 °C for 10 s, and 72 °C for 15 s; and melting curve analysis from 60 °C to 95 °C. Three replicates were included for all reactions. Primers provided by the kit were used to amplify the snRNA U6 as a housekeeping miRNA to normalize miRNA expression. The expression of each miRNA relative to U6 was calculated using the 2^−ΔΔCT^ method as described previously [71].

## Additional files

Additional file 1: **Table S1.** Top 20 most abundant miRNAs of each sample. (XLSX 11 kb)

Additional file 2: **Table S2.** DEMs between SI-10 and SC-10 samples. (XLSX 14 kb)

Additional file 3: **Table S3.** DEMs between SI-25 and SC-25 samples. (XLSX 10 kb)

Additional file 4: **Table S4.** DEMs between SI-50 and SC-50 samples. (XLSX 17 kb)

Additional file 5: **Table S5.** KEGG pathways of target genes of DEMs between the infected and control groups at 10, 25 and 50 DPI. (XLSX 43 kb)

Additional file 6: **Figure S1.** Target genes of DEMs between the infected and control groups enriched in Hematopoietic cell lineage pathway at 10, 25 and 50 DPI, respectively. (TIF 17385 kb)

Additional file 7: **Table S6.** DEMs induced by *T. gondii* at 10, 25 and 50 DPI regulating the gene expression in Hematopoietic cell lineage pathway. (XLSX 12 kb)

Additional file 8: **Figure S2.** Target genes of DEMs between SI-50 and SC-50 samples enriched in Chagas disease pathway. (TIF 11401 kb)

Additional file 9: **Table S7.** DEMs induced by *T. gondii* at 50 DPI regulating the gene expression in Chagas disease pathway. (XLSX 11 kb)

Additional file 10: **Figure S3.** Target genes of DEMs between SI-50 and SC-50 samples enriched in BCR signaling pathway. (TIF 6420 kb)

Additional file 11: **Table S8.** DEMs induced by *T. gondii* at 50 DPI regulating the gene expression in BCR signaling pathway. (XLSX 10 kb)

## Abbreviations

CSF3: Colony stimulating factor 3
DCs: Dendritic cells
DEMs: Differentially expressed miRNAs
DPI: day Post-infection
EPO: Erythropoietin
GEO: Gene Expression Omnibus
GO: Gene ontology
IFN: Interferons
ILs: Interleukins
KEGG: Kyoto Encyclopedia of Genes and Genomes
LY: Lymphocyte antigen
NK: Natural killer
RT-qPCR: Quantitative reverse transcription real-time PCR
sRNA: Small RNA
TLR: Toll-like receptor
TNF: Tumor necrosis factors
TPM: The number of transcripts per million clean tags
