# Supplementary figures and images for: Comparison of splenocyte microRNA expression profiles of pigs during acute and chronic toxoplasmosis
Source: BMC Genomics. 2019 Jan 30;20:97. doi: 10.1186/s12864-019-5458-y (PMC6354428; doi:10.1186/s12864-019-5458-y)

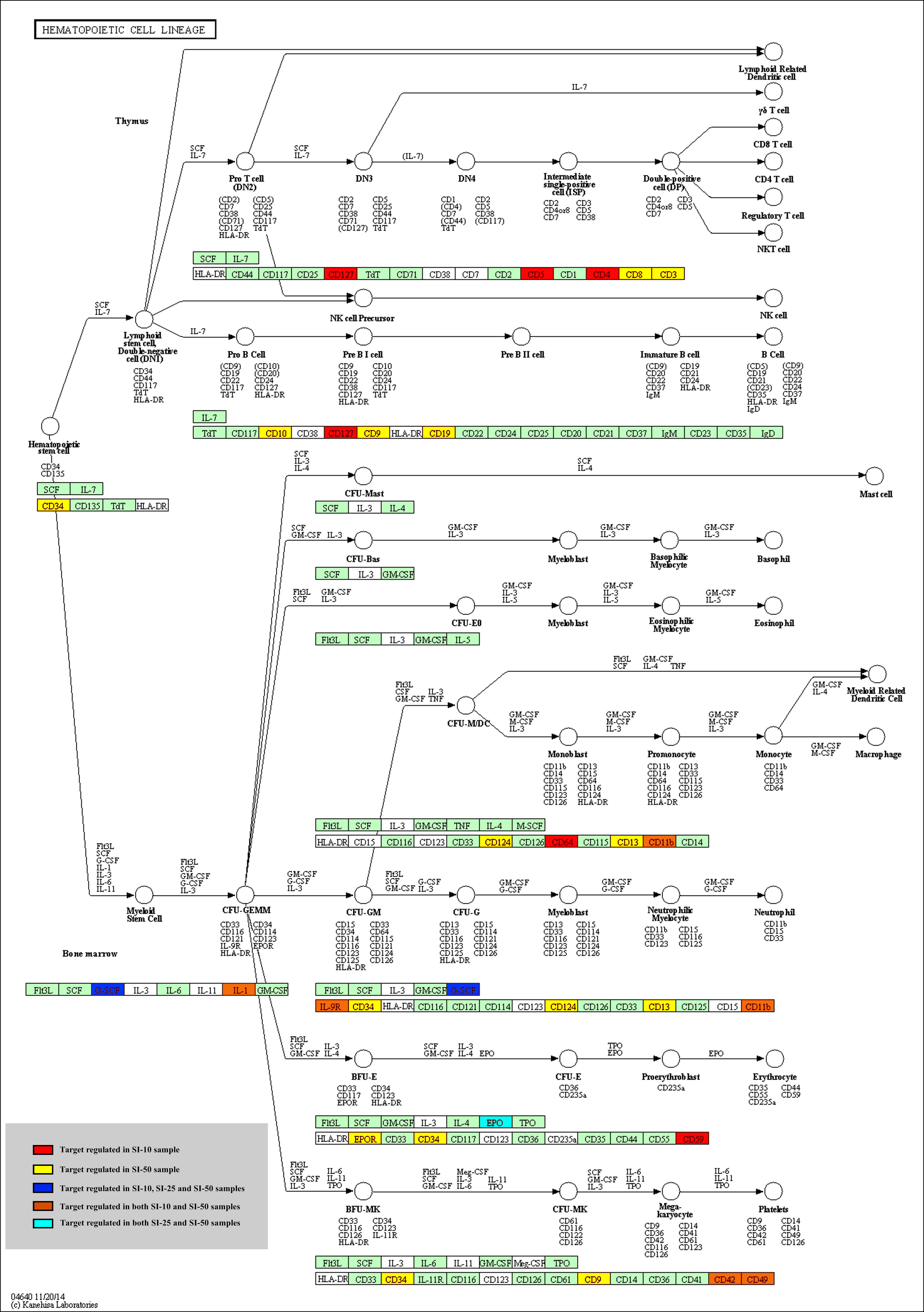

Supplement: Supplementary file 6 — Figure S1. Target genes of DEMs between the infected and control groups enriched in Hematopoietic cell lineage pathway at 10, 25 and 50 DPI, respectively. (TIF 17385 kb) [file 12864_2019_5458_MOESM6_ESM.tif]

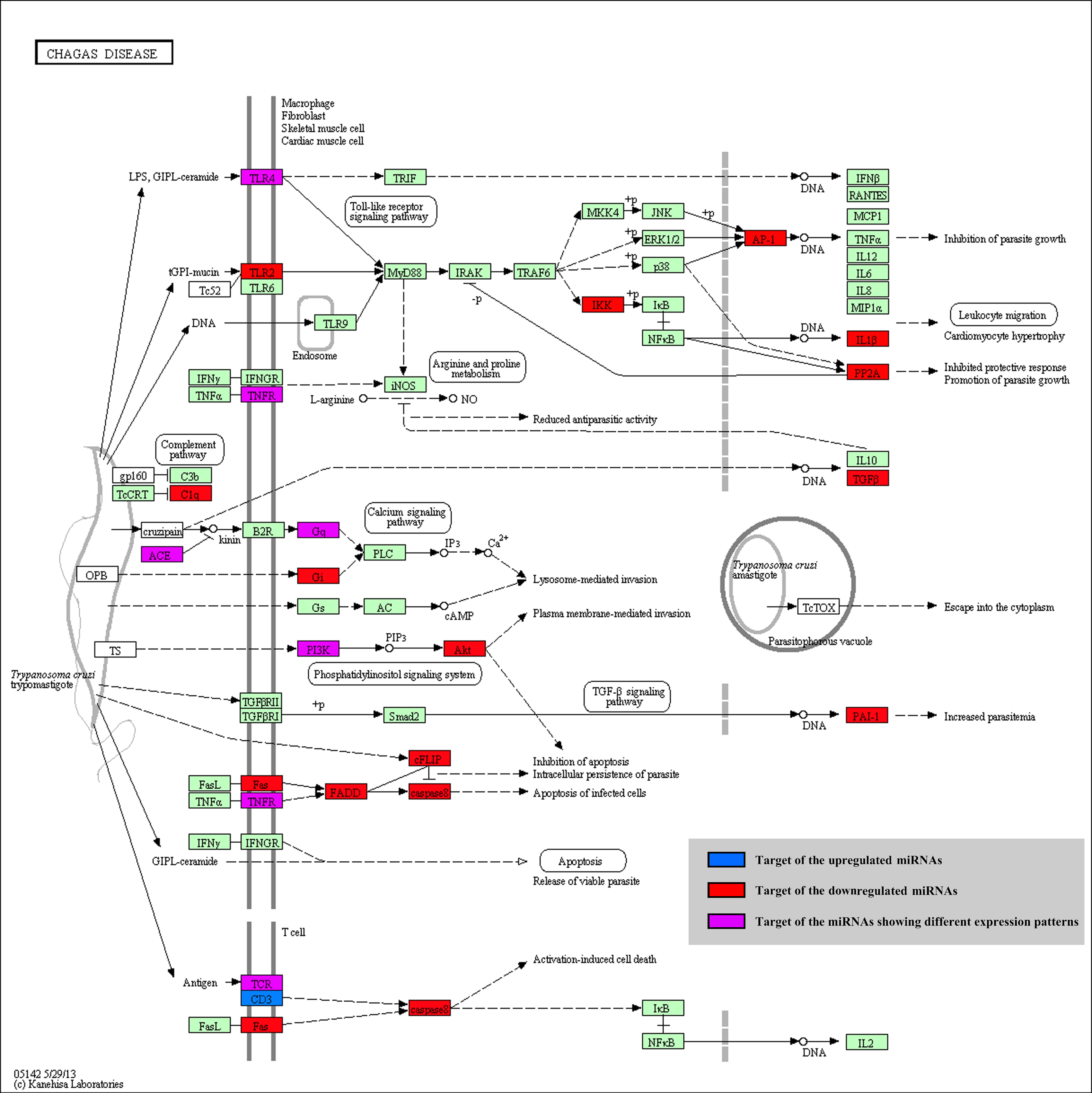

Supplement: Supplementary file 8 — Figure S2. Target genes of DEMs between SI-50 and SC-50 samples enriched in Chagas disease pathway. (TIF 11401 kb) [file 12864_2019_5458_MOESM8_ESM.tif]

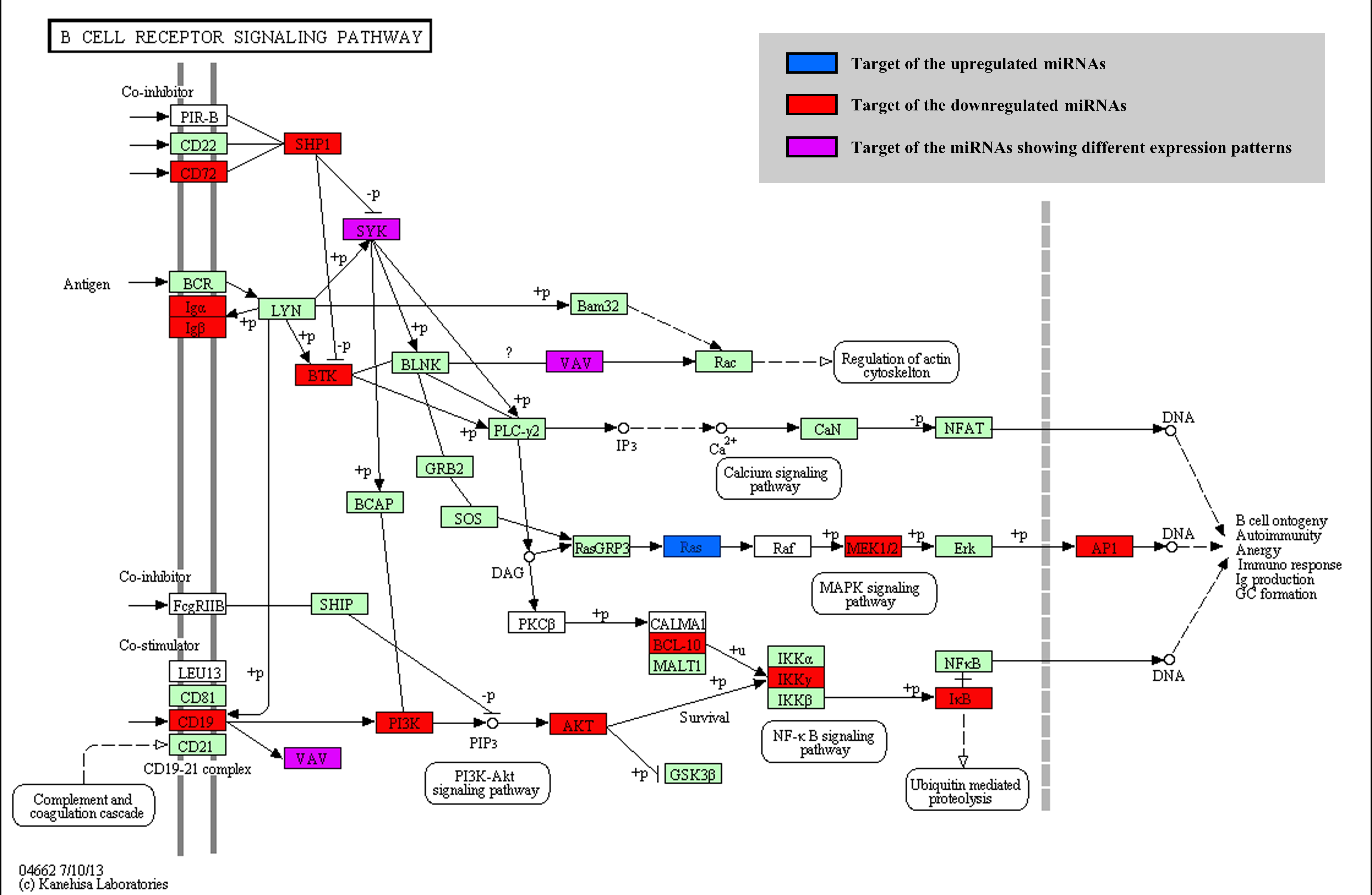

Supplement: Supplementary file 10 — Figure S3. Target genes of DEMs between SI-50 and SC-50 samples enriched in BCR signaling pathway. (TIF 6420 kb) [file 12864_2019_5458_MOESM10_ESM.tif]
